# Efficacy and safety of first-line osimertinib monotherapy versus osimertinib plus platinum-based chemotherapy or amivantamab plus lazertinib in metastatic EGFR-mutated NSCLC: an indirect comparison

**DOI:** 10.3389/fonc.2026.1802008

**Published:** 2026-05-08

**Authors:** Luna Del Bono, Andrea Ossato, Lorenzo Gasperoni, Stefano Vecchia, Luca Cancanelli, Vera Damuzzo, Stefania Gori, Roberto Tessari, Teodoro Sava, Alessandro Inno

**Affiliations:** 1 School of Specialization in Hospital Pharmacy, Department of Pharmacy, University of Pisa, Pisa, Italy; 2 Territorial Pharmaceutical Service, Azienda Unità Locale Socio-Sanitaria (ULSS) 8 Berica, Vicenza, Italy; 3 Italian Society of Clinical Pharmacy and Therapeutics, Turin, Italy; 4 Pharmaceutical Department, Unità Sanitaria Locale (USL) Toscana Centro, Prato, Italy; 5 Hospital Pharmacy Unit, Ospedale Guglielmo da Saliceto, Piacenza, Italy; 6 Hospital Pharmacy Department, Azienda Ulss 2 Marca Trevigiana, Treviso, Italy; 7 Medical Oncology, Istituto di Ricovero e Cura a Carattere Scientifico (IRCCS) Ospedale Sacro Cuore Don Calabria, Negrar di, Valpolicella, Italy; 8 Hospital Pharmacy, Istituto di Ricovero e Cura a Carattere Scientifico (IRCCS) Ospedale Sacro Cuore Don Calabria, Negrar di, Valpolicella, Italy

**Keywords:** amivantamab, EGFR-mutated NSCLC, first-line therapy, indirect treatment comparison, lazertinib, network meta-analysis, osimertinib, reconstructed individual patient data

## Abstract

**Background:**

Osimertinib (Osi) has long represented the standard first-line treatment for metastatic EGFR-mutated non-small cell lung cancer. Recently, two randomized phase 3 trials demonstrated that osimertinib plus platinum-based chemotherapy (Osi+CT) and amivamtamab plus lazertinib (Ami+Laz) improve outcomes compared with Osi alone, although no direct comparison between these regimens are available. This study aimed to indirectly compare progression-free survival (PFS) and overall survival (OS).

**Materials and methods:**

Three randomized clinical trials (FLAURA, FLAURA-2, and MARIPOSA) were included following PRISMA guidelines. Individual patient data (IPD) were reconstructed from Kaplan-Meier curves using the IPDfromKM algorithm. Hazard ratios (HR) with 95% confidence intervals (CI) and restricted mean survival time (RMST) were calculated for PFS and OS. Subgroup analyses were performed for patients with central nervous system (CNS) metastases and by EGFR mutation subtypes. A network meta-analysis was conducted to evaluate adverse drug reactions.

**Results:**

In the indirect comparison, Osi+CT and Ami+Laz showed no statistical difference in terms of PFS (HR 0.79, 95% CI 0.61-1.04), with PFS-RMST estimates at 30 months numerically longer with Osi+CT (22.53 vs 20.59 months). No statistically significant differences in OS were observed between regimens (HR 0.95, 95% CI 0.73–1.22). Subgroup PFS analyses according to baseline CNS metastases and EGFR mutation subtypes showed no statistically conclusive differences between regimens. Safety profiles varied: Osi+CT was associated with higher rates of grade ≥ 3 hematologic and gastrointestinal toxicities, while Ami+Laz was linked to higher rates of dermatologic events, infusion-related reactions, hypoalbuminemia, and peripheral edema.

**Conclusions:**

Despite the inherent limitations of indirect comparisons, these findings suggest that Osi+CT and Ami+Laz provide comparable survival benefits, while distinct toxicity profiles may influence therapeutic decision-making in conjunction with patients’ clinical characteristics and preferences.

## 1 Introduction

Osimertinib has long represented the standard first-line treatment for metastatic non-small cell lung cancer (NSCLC) with common activating epidermal growth factor receptor (EGFR) mutations, including exon 19 deletions and the exon 21 L858R substitution (1). In the FLAURA trial (2, 3), osimertinib achieved a median progression-free survival (PFS) of 18.9 months and a median overall survival (OS) of 38.6 months, establishing its role as the preferred first-line option. Importantly, osimertinib also demonstrated substantial intracranial activity, with an 18-month intracranial PFS rate of 54% in patients with central nervous system (CNS) metastases (4, 5).

Despite these favorable outcomes, acquired resistance to osimertinib invariably develops. Both on-target alterations—such as EGFR amplification or secondary mutations—and off-target mechanisms involving bypass pathway activation (e.g., MET or HER2) or histologic transformation to small-cell lung cancer (SCLC) contribute to disease progression (6–9). These mechanisms have prompted the development of novel therapeutic strategies aimed at prolonging osimertinib benefit or delaying resistance, including combinations with chemotherapy, bispecific EGFR/MET antibodies, and antibody–drug conjugates (ADCs) (10, 11).

Among these strategies, the most advanced in clinical development are the combination of osimertinib with platinum-based chemotherapy, and the combination of the third-generation TKI lazertinib with the bispecific anti-EGFR/MET antibody amivantamab. The phase 3 randomized FLAURA-2 trial assessed the addition of platinum-based chemotherapy to osimertinib, demonstrating superior efficacy compared with osimertinib alone (12–14). Similarly, the phase 3 randomized MARIPOSA trial tested the combination of lazertinib with the anti-EGFR/MET bispecific antibody amivantamab, also showing superiority over standard osimertinib (15, 16). However, these regimens have not been directly compared with each other.

To address this gap, we performed an indirect comparison of first-line osimertinib monotherapy, osimertinib plus platinum-based chemotherapy, and amivantamab plus lazertinib. We reconstructed individual patient data (IPD) from the FLAURA, FLAURA 2 and MARIPOSA trials using the IPDfromKM algorithm, which enables extraction of patient-level time-to-event data from published Kaplan-Meier (KM) curves (17–19). This approach supports standardized cross-trial comparisons based on a shared comparator arm and has been widely used for indirect analyses across multiple medical fields (17).

## 2 Materials and methods

### 2.1 Literature search

A systematic search of the PubMed database was performed to identify randomized controlled trials (RCTs) relevant to this analysis. The last search was performed on 18th January 2026. The search strategy was: [(“Non-Small Cell Lung Cancer”[Mesh] OR “NSCLC” OR “lung adenocarcinoma”) AND (“EGFR mutations” OR “EGFR-mutant” OR “EGFR-positive” OR “EGFR”) AND (“first-line treatment” OR “frontline therapy” OR “initial therapy” OR “untreated”) NOT adjuvant NOT neoadjuvant]. The study selection followed the PRISMA guidelines (20). Inclusion criteria were: (a) randomized phase III design; (b) first-line treatment of metastatic EGFR-mutated NSCLC; (c) inclusion of osimertinib as a comparator arm; (d) availability of PFS and OS data; (e) publication of Kaplan-Meier (KM) survival curves. The FLAURA trial was specifically included to provide the osimertinib arm considered standard of care, and for historical context through its control arm of earlier-generation EGFR-TKIs. For each study, we extracted the total number of patients enrolled and the number of events (disease progression or death). When multiple publications of the same trial were available, the most recent and complete version was selected to avoid duplication. Accordingly, PFS and OS KM curves of FLAURA, FLAURA 2 and MARIPOSA trials were obtained from different publications, as OS results were reported later than. For PFS we used datasets from Soria et al. (FLAURA), Planchard et al. (FLAURA 2), and Cho et al. (MARIPOSA) (2, 12, 15). For OS, we used Ramalingam et al. (FLAURA), Valdiviezo et al. (FLAURA 2) and Yang et al. (MARIPOSA) (3, 13, 16).

### 2.2 Reconstruction of individual patient data

To compare the efficacy of the evaluated treatments, IPD were reconstructed from the KM curves of the selected randomized clinical trials (RCTs) using the IPDfromKM algorithm. This method has been formally validated, demonstrating excellent concordance between reconstructed and original patient-level data, particularly when accounting for censored observations (18, 19). First, KM curves were digitized using WebPlotDigitizer (version 4.7; accessed online at https://apps.automeris.io/wpd/on January 18, 2026). The extracted X and Y coordinates, together with the total number of patients and events, were subsequently entered into the IPDfromKM software (version 1.2.3.0, last updated on March 22, 2022). The software generated reconstructed IPD for each RCT arm, providing survival times (defined as the time from enrollment to the last follow-up) and event status categorized as alive/censored or dead/progressed. Osimertinib was selected as the common comparator across trials, and patients treated with osimertinib were pooled to constitute as the reference control for indirect comparisons.

### 2.3 Study design and data analysis

In our analysis, three regimens were considered: a) osimertinib in combination with chemotherapy (Osi+CT), b) amivantamab in combination with lazertinib (Ami+Laz), c) osimertinib monotherapy (Osi). The primary objective was to identify the treatment associated with the greatest PFS and OS. PFS and OS KM curves were analyzed for each treatment and compared both across treatment groups and against a pooled control curve representing patients treated with Osi, which served as the common comparator.

The MARIPOSA trial also included a lazertinib monotherapy arm, which was intended solely to explore the individual contribution of Lazertinib within the Ami+Laz combination. As this arm was not submitted for the regulatory approval and is not clinically relevant in current practice, it was excluded from the present analysis.

To ensure consistency within the anchored indirect comparison framework, Osi monotherapy was used as the common reference comparator across all analyses. Accordingly, for the FLAURA trial, outcomes were expressed relative to the Osi arm to align with the design of FLAURA-2 and MARIPOSA. In the safety analyses, this approach allowed the 1st-generation EGFR-TKI arm to be evaluated relative to Osi, ensuring consistency across all comparisons.

Prespecified subgroup analyses were performed for PFS in patients with CNS metastases at baseline and according to EGFR mutation subtype (exon 19 deletion and L858R). PFS and OS were analyzed using Cox proportional hazards model, and results were expressed as hazard ratios (HRs) with corresponding 95% confidence intervals (CIs). The homogeneity of the pooled control groups across trials was evaluated using the likelihood ratio tests and concordance statistics, in line with standard methods for assessing between-studies heterogeneity. Indirect comparisons among active treatments were conducted using a Cox model to allow pairwise estimation across all treatment combinations.

Survival estimates were additionally quantified using Restricted Mean Survival Time (RMST) (21). KM curves were truncated at 30 months for PFS-RMST calculation and 47 months for OS-RMST, corresponding to the minimum follow-up durations available for each endpoint across the included RCTs. All statistical analyses were performed using R software (version 4.3.2).

Finally, the most frequently reported adverse drug reactions (ADRs), both of any grade and grade ≥3, were extracted from the included studies and tabulated by treatment group. A frequentist network meta-analysis based on a random effect model was conducted using the MetaInsight software (22) to estimate the relative risk (RR) of each ADR among different treatments, adopting Osi as the reference comparator. Binary meta-analysis and forest plot has been generated using and Meta-Mar (v4.0.2) software.

## 3 Results

### 3.1 Indirect comparison: efficacy analysis

**Figure 1:**
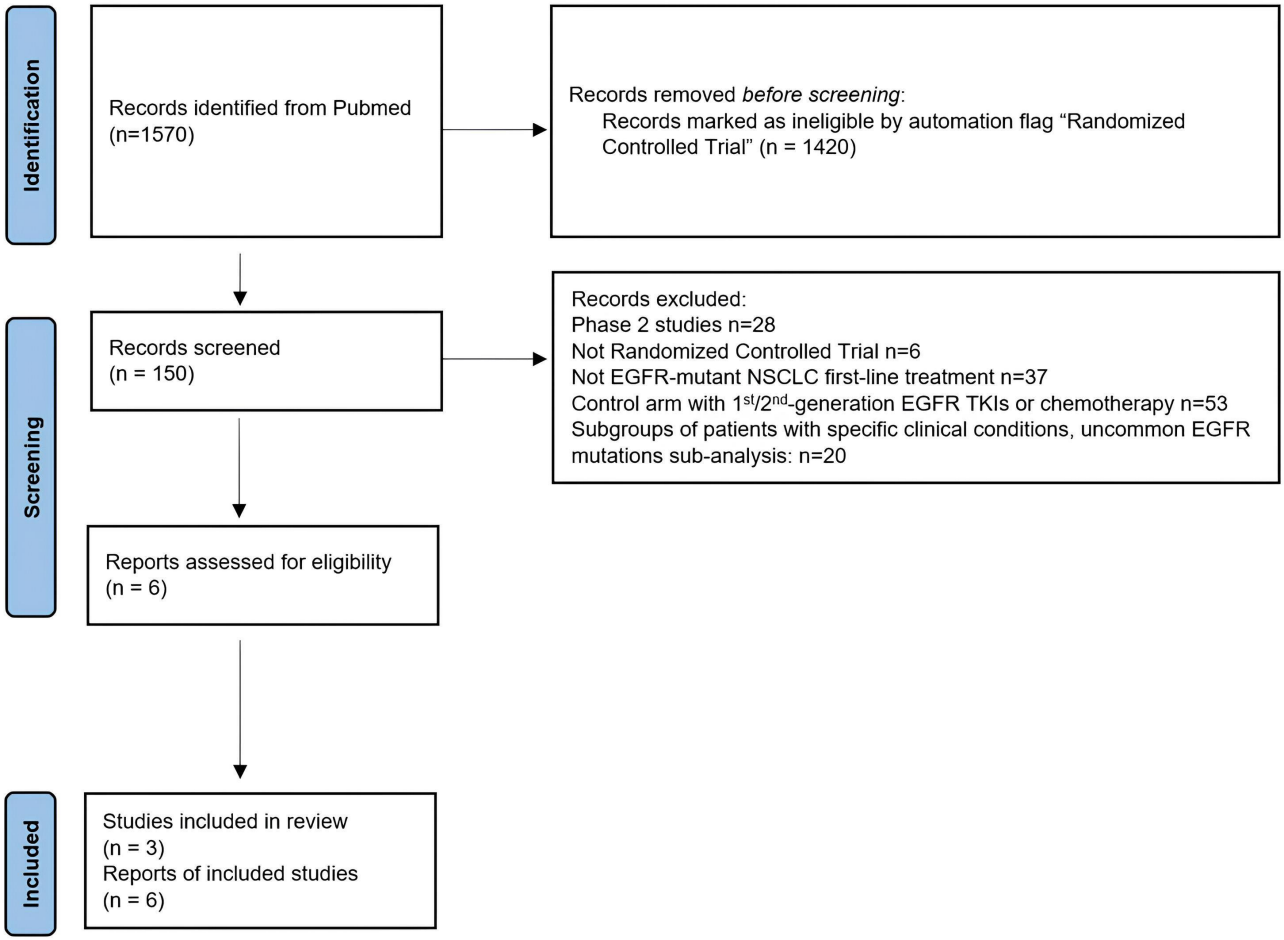
PRISMA flowchart of the process of trial selection. EGFR, epidermal growth factor receptor; TKI, tyrosine kinase inhibitor; NSCLC, non-small cell lung cancer.

The literature search identified 1570 records which were screened according to the predefined inclusion and exclusion criteria to identify randomized clinical trials (RCTs) evaluating first-line EGFR inhibitors for advanced or metastatic NSCLC. The study selection process is summarized in **Figure 1**, in accordance to PRISMA guidelines. Three RCTs were ultimately identified for our indirect comparison analysis based on PFS and OS endpoints.

The FLAURA trial compared osimertinib with first-generation EGFR-TKIs (gefitinib or erlotinib) (2, 3). The FLAURA-2 trial assessed the benefit of adding platinum-based chemotherapy to osimertinib (Osi+CT) (12–14), whereas the MARIPOSA trial compared osimertinib with the combination of amivantamab and lazertinib (Ami+Laz) (15, 16). All included studies contained an osimertinib (Osi) treatment arm that was used as the common comparator for the indirect comparison.

**Table 1** summarizes the main clinical and demographic characteristics of patients enrolled in the included trials.

**Table 1:** Clinical characteristics of patients included in the analysis Median follow-up time, median PFS and median OS are expressed in months.

Trial	Treatments arms	N. of patients	% Pt. Smoking history	% Pt. Asian	% Pt. CNS metastasis BL	% Pt. Exon 19 del	% Pt. L858R mut	Median PFS	HR for PFS (95%CI)	Median OS	HR for OS (95%CI)
FLAURA (2, 3)	Osimertinib vs 1^st^-gen EGFR-TKI	279 277	35% 37%	62% 62%	19% 23%	63% 63%	37% 37%	18.9 10.2	0.46 (0.37-0.57)	38.6 31.8	0.80 (0.64-1.00)
FLAURA 2 (12–14)	Osimertinib + CT vs Osimertinib	279 278	33% 35%	64% 63%	42% 40%	61% 60%	38% 38%	25.5 16.7	0.62 (0.49-0.79)	NR 36.7	0.75 (0.57-0.97)
MARIPOSA (15, 16)	Ami+Laz vs Osimertinib	429 429	30% 31%	58% 59%	41% 40%	60% 60%	40% 40%	23.7 16.6	0.70 (0.58-0.85)	NR 36.7	0.75 (0.61-0.92)

Overall, patient populations were largely homogeneous in terms of smoking history, ethnicity and the proportion of exon 19 deletions and L858R mutations. However, the FLAURA trial enrolled a lower proportion of patients with baseline central nervous system (CNS) metastases compared to FLAURA-2 and MARIPOSA. To account for this difference, a heterogeneity analysis was conducted to assess whether the cohorts treated with the same agent (Osi) behaved comparably across trials. No significant heterogeneity was detected for either PFS (likelihood ratio test = 0.47; df=2; p = 0.80) or OS (likelihood ratio test = 0.75; df=2; p = 0.70), supporting the overall comparability of the studies, despite numerical differences in baseline characteristics. KM curves for PFS and OS of the control arms are reported in **Supplementary Figures 1A, B**, respectively).

**Figure 2:**
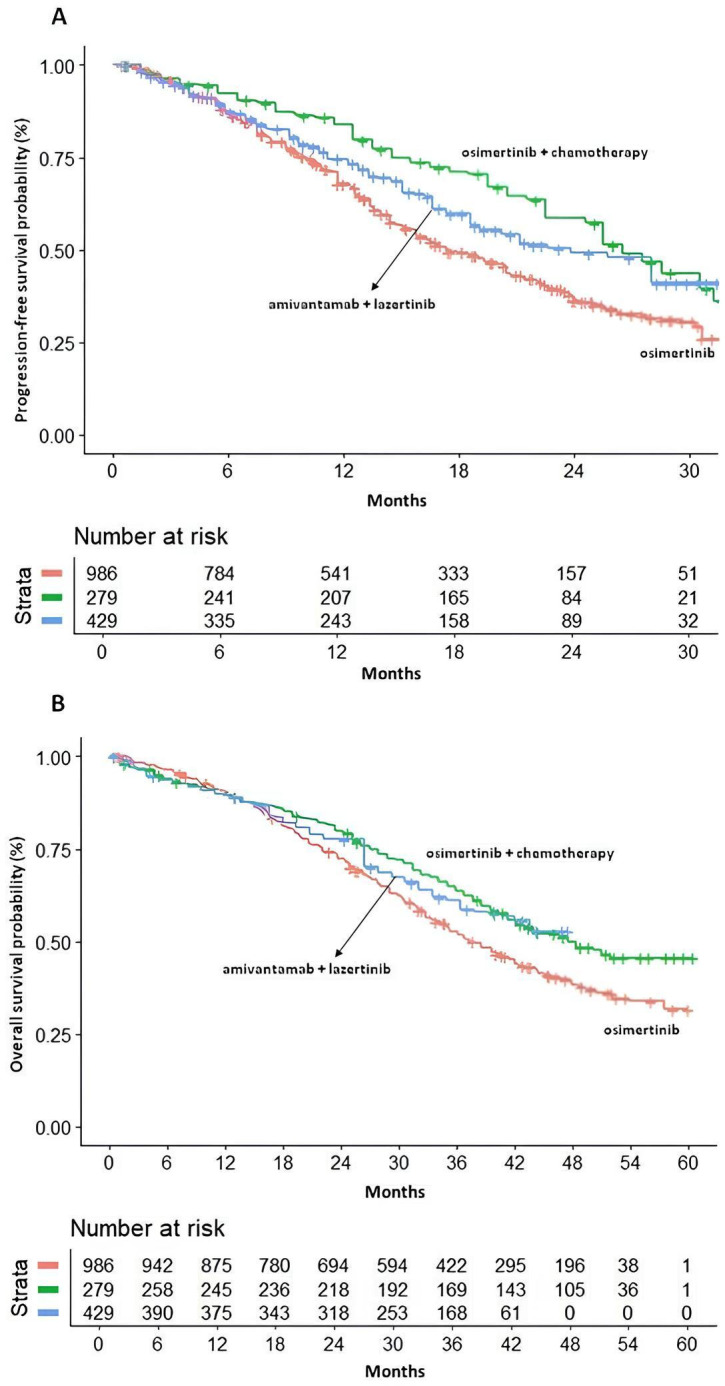
**(A)** PFS of first-line treatment options compared to osimertinib controls. After reconstruction of individual patient data from three trials, the following PFS KM curves were generated: Osi (n = 986; 3 cohorts (2, 12, 15); in red); Osi+CT (n = 279 from FLAURA 2 study (12); in green); and Ami+Laz (n = 429 from MARIPOSA study (15); in blue). **(B)** OS of first-line treatment options compared to osimertinib controls. After reconstruction of individual patient data from three trials, the following OS KM curves were generated: Osi (n = 986; 3 cohorts (3, 14, 16); in red); Osi+CT (n = 279 from FLAURA 2 study (14); in green); and Ami+Laz (n = 429 from MARIPOSA study (16); in blue). n, number of patients.

After confirming trial comparability, efficacy outcomes of the three different treatments were assessed. PFS and OS KM curves for each inhibitor are shown in **Figures 2A, B**, respectively.

The combination of Osi+CT and Ami+Laz resulted in a significant PFS benefit compared with osimertinib alone (respectively HR 0.58, 95% CI 0.47–0.71 and HR 0.73, 95% CI 0.61–0.87). In the indirect comparison, Osi+CT showed a numerical PFS advantage over Ami+Laz, although the difference did not reach statistical significance (HR 0.79, 95% CI 0.61–1.04). RMST analysis at 30 months was consistent with these results, with a PFS-RMST of 22.53 months (95% CI 21.4-23.67) for Osi+CT, corresponding to an approximately 2-month longer PFS-RMST compared with Ami+Laz (20.59 months, 95% CI 19.53-21.65).

In the OS analysis, both Osi+CT (HR 0.74, 95% CI 0.61–0.89) and Ami+Laz (HR 0.78, 95% CI 0.65–0.93) were associated with improved outcomes compared with Osi alone. No significant OS difference was observed between Osi+Ct and Ami+Laz (HR 0.95, 95% CI 0.73–1.22). Consistently, RMST analysis at 47 months showed comparable OS-RMST estimates between Osi+CT (36.43 months 95% CI 34.75-38.15) and Ami+Laz. (35.66 months 95% CI 34.25 - 37.1).

**Figure 3:**
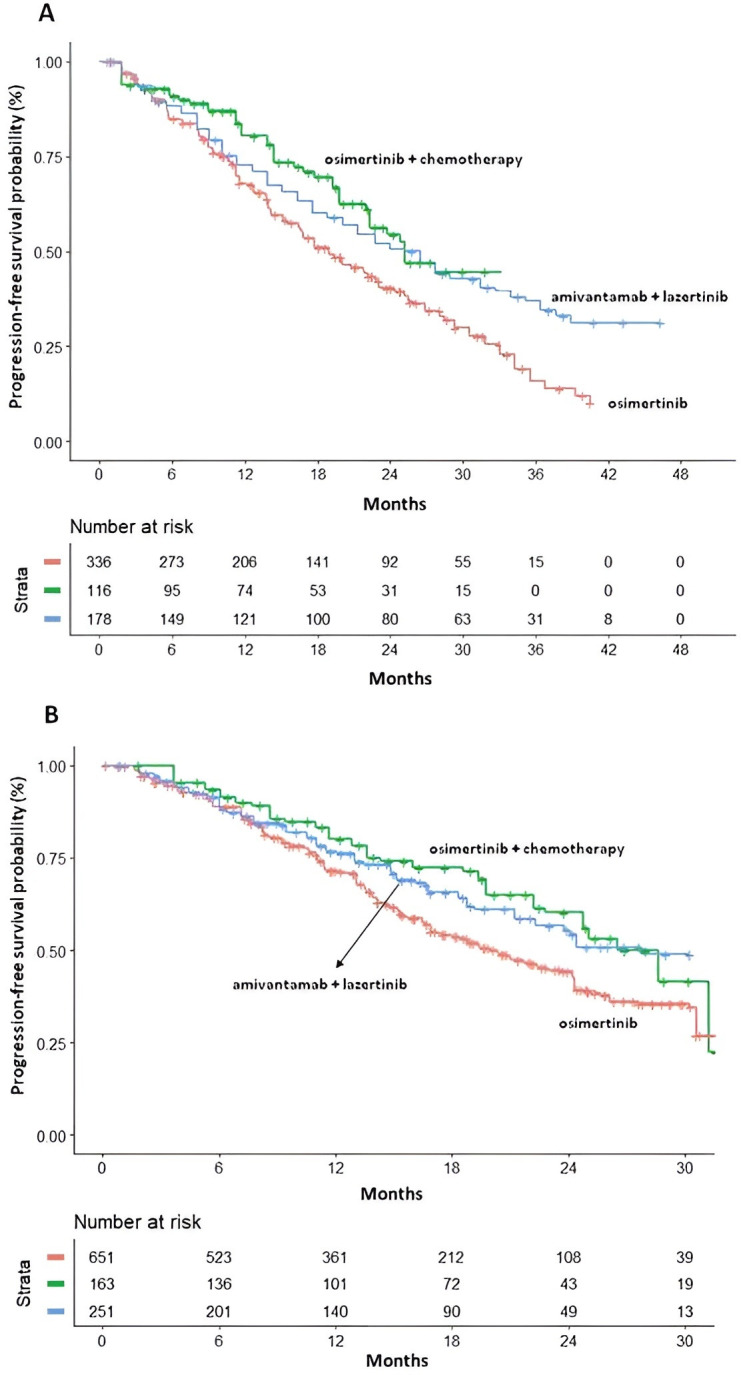
PFS in patients with **(A)** and without **(B)** CNS metastasis at baseline. PFS KM curves are respectively reported: Osi (n = 336 and n=651; 3 cohorts (2, 12, 15); in red); Osi+CT (n = 116 and n=163 from FLAURA 2 study (12); in green); and Ami+Laz (n = 178 and n=251 from MARIPOSA study[15]; in blue). n, number of patients.

Given the availability of subgroup-specific KM curves, PFS analyses were conducted on patients with and without baseline CNS metastases and according to the EGFR mutation subtype. **Figure 3** reports PFS curves stratified for CNS involvement.

In patients with baseline CNS metastases, both Osi+CT (HR 0.69, 95% CI 0.50–0.93) and Ami+Laz (HR 0.65, 95% CI 0.52–0.83) improved PFS compared with Osi alone. No significant difference was observed between Osi+CT and Ami+Laz in the indirect comparison (HR 1.05, 95% CI 0.71–1.55). At 30 months, PFS-RMST for Osi+CT was 22.09 months (95% CI 20.23-23.95) corresponding to an absolute gain of 3.28 months compared with Osi alone, and 1.35 months compared with Ami+Laz (18.81 months, 95% CI 17.7-19.91, and 20.75 months, 95% CI 19.22-22.27, respectively).

In patients without CNS metastases, both Osi+CT (HR 0.71; 95% CI 0.54–0.92) and Ami+Laz (HR 0.73; 95% CI 0.57–0.92) were superior to Osi in terms of PFS, with no significant difference between the two combination strategies (HR 0.97, 95% CI 0.68–1.4). RMST analysis at 30 months showed a gain of 3.1 months for Osi+CT (22.55 months, 95% CI 20.99-24.11) and 2.19 months for Ami+Laz (21.65 months, 95% CI 20.27-23.03) compared with Osi alone, resulting in an absolute difference of 0.9 months in favor of Osi+CT.

**Figure 4:**
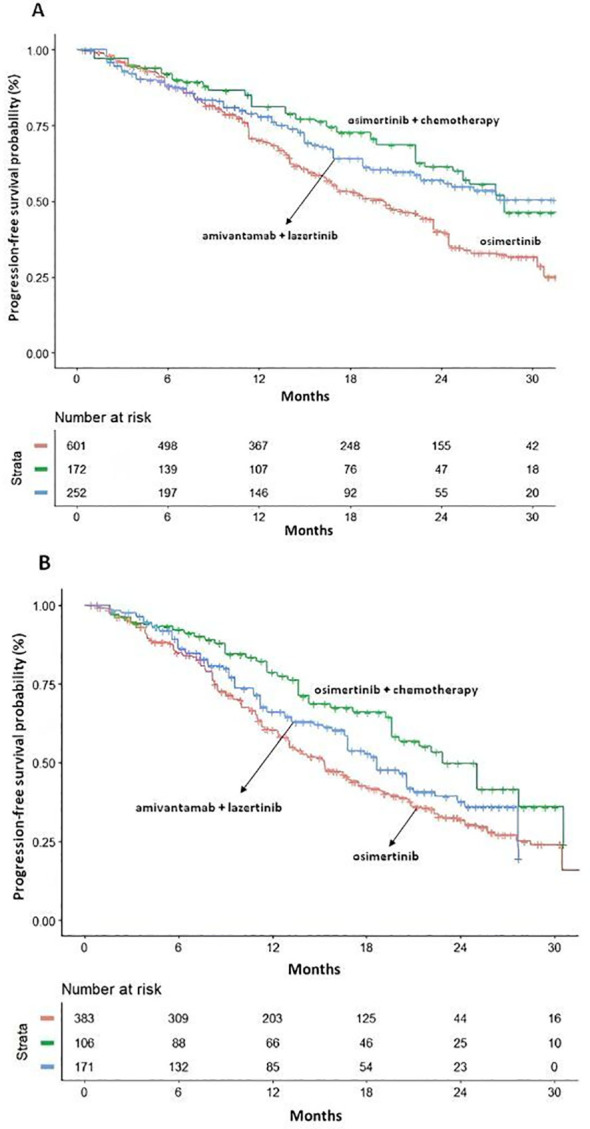
PFS of EGFRi in patients with Exon19 deletion **(A)** or with L858R mutation in exon 21 **(B)**. PFS KM curves are respectively reported: osimertinib (n=601 and n=383; 3 cohorts (2, 12, 15); in red); Osi+CT (n =172 and n=106 from FLAURA 2 study (12); in green); and Ami+Laz (n=252 and n=171 from MARIPOSA study (15); in blue). n, number of patients.

In **Figure 4**, KM curves for PFS in patients harboring exon19 deletion or L858R mutation in exon21 are reported.

In patients with exon 19 deletion and L858R mutation both Osi+CT (exon 19: HR 0.56; 95% CI 0.42–0.74; L858R: HR 0.60; 95% CI 0.44–0.82) and Ami+Laz (exon 19: HR 0.65; 95% CI 0.51–0.82; L858R: HR 0.84; 95% CI 0.65–1.08) were superior to Osi in terms of PFS.

Across mutation subgroups, Osi+CT showed a numerical PFS advantage over Ami+Laz in both patients with exon 19 deletion and L858R mutation, although no significantly statistical differences were observed. The magnitude of this numerical advantage appeared more pronounced in the L858R subgroup (exon 19: HR 0.87, 95% CI 0.6–1.25; L858R: HR 0.71, 95% CI 0.48–1.06). In the exon 19 deletion subgroup, the RMST analysis at 30 months showed a PFS-RMST gain of 3.8 months for Osi+CT (22.94 months, 95% CI 21.4-24.47) and 2.54 months for Ami+Laz (21.69 months, 95% CI 20.29-23.09) compared with Osi alone, corresponding to an absolute difference of 1.25 months in favor of Osi+CT. In patients with L858R-mutation, Osi+CT was associated with a numerically longer PFS-RMST at 30 months compared with Ami+Laz (Osi+CT 19.94 months 95%CI 18.25-21.63; Ami+Laz 17.8 months 95%CI 16.36-19.23), corresponding to an absolute difference of 2.14 months.

### 3.2 Adverse drug reactions analysis

The incidence of any grade adverse drug reactions (ADRs) was comparable between Osi+CT and Ami+Laz, whereas Osi was associated with a significantly lower incidence of grade ≥3 ADRs compared with both combination regimens (RR = 0.43, 95%CI 0.35 - 0.53 compared to Osi+CT and RR = 0.57, 95%CI 0.50 - 0.64 compared to Ami+Laz). Grade ≥3 ADRs were more common in patients treated with Osi+CT than in those receiving Ami+Laz (RR = 0.75, 95%CI 0.59 - 0.96). Results are reported in **Supplementary Figure 2**.

**Figure 5:**
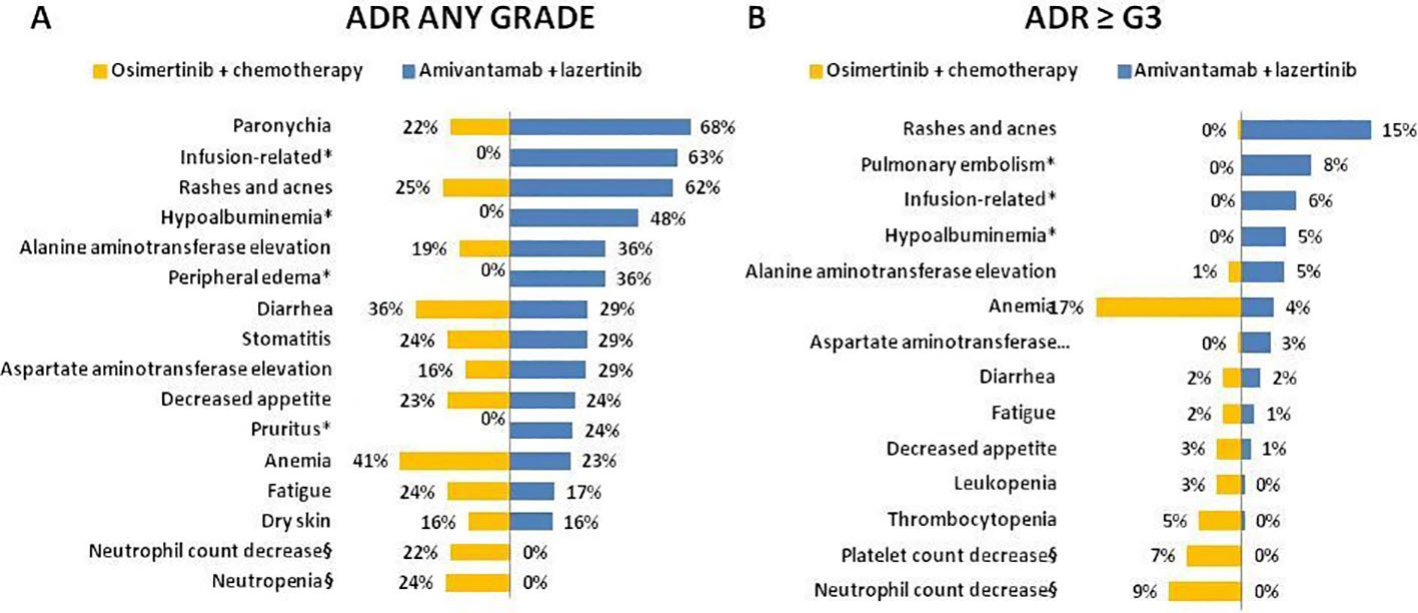
Incidence of any grade **(A)** and severe ADR (≥G3) **(B)** in FLAURA 2 and MARIPOSA studies are reported in orange and blue bars, respectively. *Not reported in FLAURA2 study, § not reported in MARIPOSA study.

Overall, toxicity profiles differed substantially between regimens (**Figure 5**). Osi+CT was primarily associated with anemia, diarrhea and cutaneous reactions (rash, acne), whereas. Ami+Laz was characterized by cutaneous ADR (paronychia, rash and acne) hepatic toxicity and infusion-related reactions.

Severe ADRs in the Osi+CT group were mainly related to chemotherapy-associated toxicities, including fatigue, anemia, neutropenia and thrombocytopenia. Grade ≥3 ADRs occurring in patients treated with Ami+Laz mainly consisted of rash, acne, pulmonary embolism, infusion-related reactions, and hypoalbuminemia.

**Figure 6:**
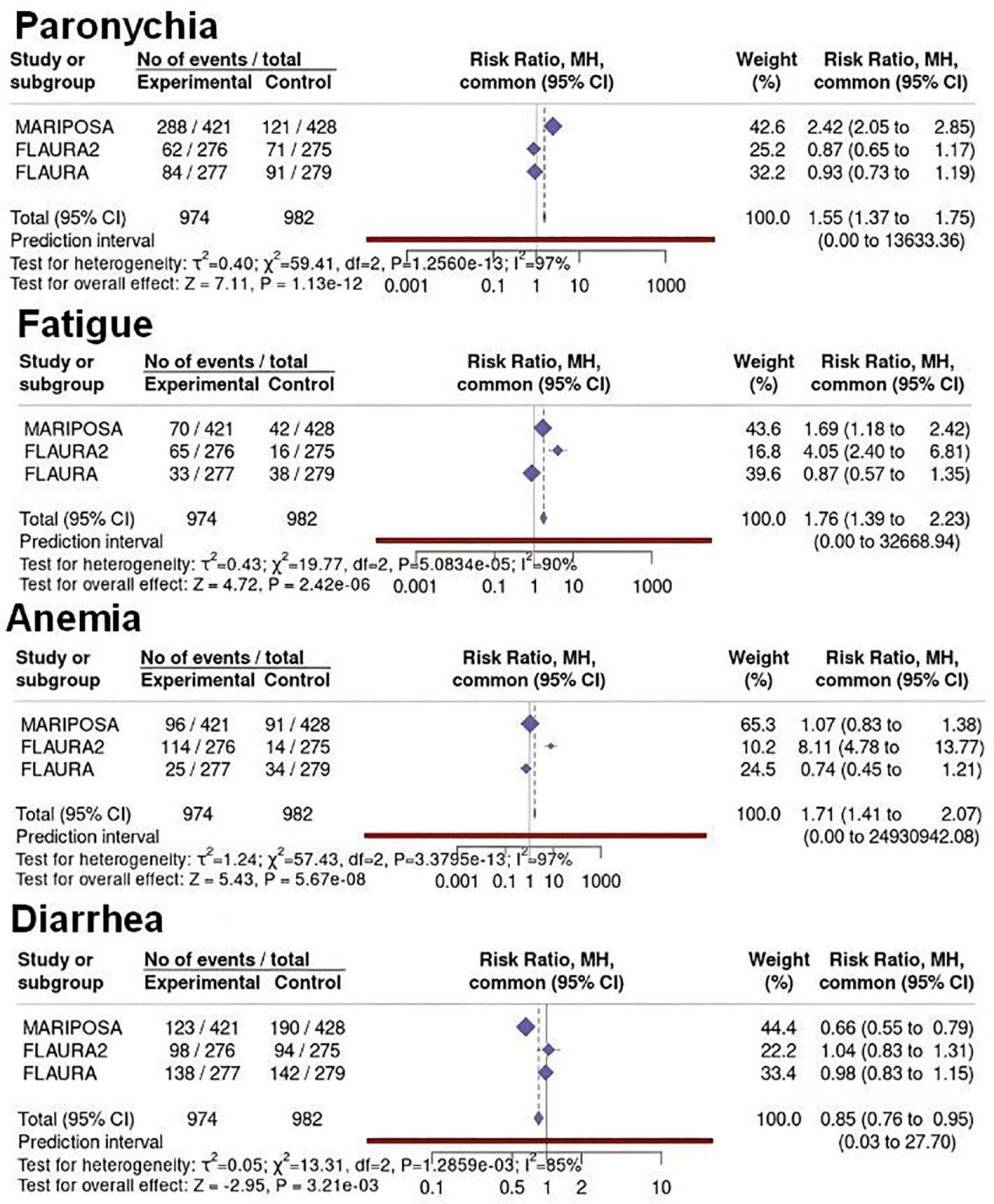
Binary meta-analysis comparing incidence of any grade ADR in patients treated with Ami+Laz (MARIPOSA Study), Osi+CT (FLAURA2 Study), and 1^st^-generation EGFR-TKI (FLAURA Study) compared to Osi monotherapy. Risk ratio estimates with 95%CI and crude rates in the active and control arms are reported for paronychia, fatigue, anemia and diarrhea. Osi monotherapy was used as the reference comparator in all analyses. For the FLAURA Study, risk ratios are expressed as 1st-generation EGFR-TKIs relative to Osi to ensure consistency with the other trials.

**Figure 7:**
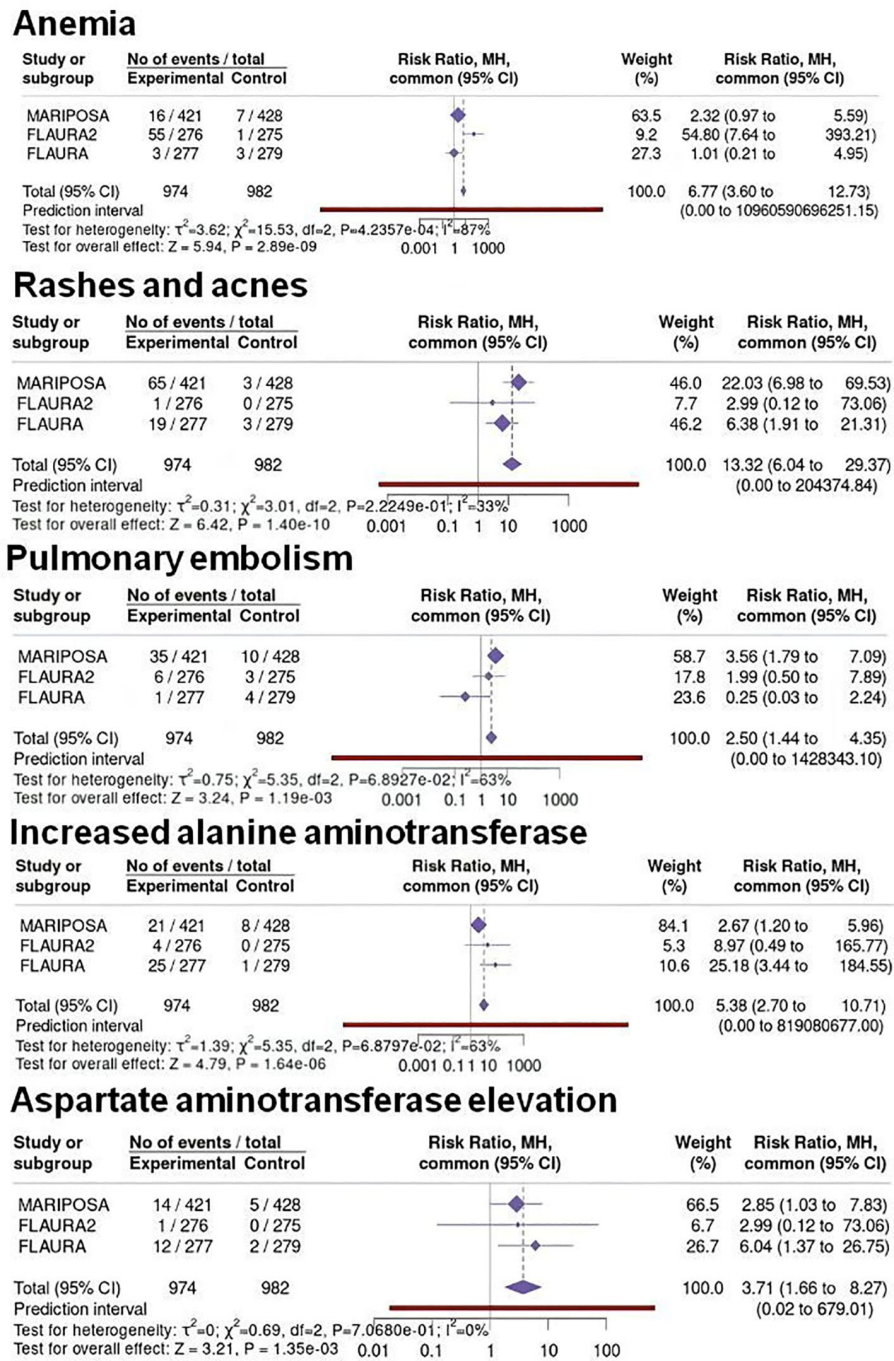
Binary metanalysis comparing incidence of grade ≥3 ADR in patients treated Ami+Laz (MARIPOSA Study), Osi+CT (FLAURA2 Study), and 1^st^-generation EGFR-TKI (FLAURA Study) compared to Osi monotherapy. Risk ratio estimates with 95%CI and crude rates in the active and control arms are reported for anemia, rashes and acnes, pulmonary embolism, increased alanine aminotransferase, and aspartate aminotransferase elevation. Osi monotherapy was used as the reference comparator in all analyses. For the FLAURA Study, risk ratios are expressed as 1st-generation EGFR-TKIs relative to Osi to ensure consistency with the other trials.

Network meta-analyses were performed using Osi monotherapy as the reference comparator across all analyses. For the FLAURA trial, results were expressed as the risk associated with 1st-generation EGFR-TKIs relative to Osi, ensuring consistency with the reference framework adopted in FLAURA-2 and MARIPOSA. The results of the network meta-analyses are shown in **Figures 6** and **7**.

Compared with Osi+CT, Ami+Laz was associated with a significantly lower risk of fatigue (RR = 0.42, 95%CI 0.22 - 0.79), anemia (RR = 0.13, 95%CI 0.07 - 0.24), and diarrhea (RR = 0.63, 95%CI 0.47 - 0.85), whereas Osi+CT showed a lower incidence of paronychia (RR = 0.36, 95%CI 0.26 - 0.50) and dry skin (RR = 0.64, 95%CI 0.40 - 1.03).

Regarding severe (grade ≥ 3) ADR, the incidence of anemia (RR = 0.04, 95%CI 0 - 0.37) were lower in the Ami+Laz group compared to Osi+CT. On the other hand, the risk of developing rashes and acne (RR = 0.14, 95%CI 0 – 4.05) and pulmonary embolism (RR = 0.56, 95%CI 0.12 – 2.51) was significantly lower in the Osi+CT group than in the Ami+Laz group. Some cases of severe hepatic toxicities were reported in both combination regimens, with Ami+Laz having a trend towards higher incidence of alanine and aspartate aminotransferase elevation.

## 4 Discussion

Osimertinib has long represented the standard of care for patients with metastatic NSCLC harboring common EGFR mutations (1–3). Recently, two combination strategies (Osi+CT and Ami+Laz) have independently demonstrated superiority over Osi monotherapy in randomized phase III trials (12, 15). However, in the absence of direct head-to-head comparisons, uncertainty remains regarding the relative efficacy and safety of these regimens, providing the rationale for indirect comparative analyses.

In this indirect comparison based on reconstructed individual patient data, no statistically significant differences in PFS were observed between Osi+CT and Ami+Laz (HR 0.79, 95% CI 0.61–1.04). However, both the point estimate of the hazard ratio and the PFS-RMST analysis at 30 months (approximately 2-month longer PFS-RMST with Osi+CT) suggest a potential numerical advantage for Osi+CT in disease control. These findings should be interpreted as hypothesis-generating rather than as evidence of equivalence.

A descriptive inspection of the PFS curves suggests an early separation in favor of Osi+CT, emerging within the first three months, followed by convergence at later time points. Although these observations should be interpreted cautiously given the indirect nature of the comparison, they raise the hypothesis that the two strategies may be associated with different temporal patterns of disease control.

From a biological perspective, resistance to first-line osimertinib is heterogeneous and frequently involves off-target bypass pathways, most notably MET amplification, in addition to on-target EGFR alterations (6–9). Molecular analyses from FLAURA-2 indicate that the addition of platinum-pemetrexed to osimertinib does not substantially alter the overall spectrum of acquired resistance mechanisms compared with osimertinib alone, with MET-driven resistance remaining a relevant pathway at progression (23). These findings suggest that chemotherapy may primarily contribute to reducing the impact of early resistance mechanisms, potentially explaining the early separation of PFS curves observed with Osi+CT.

Conversely, exploratory analyses from the MARIPOSA trial suggest that upfront dual EGFR and MET inhibition with amivantamab plus lazertinib may mitigate the clinical impact of MET-driven resistance mechanisms (24). Sustained MET blockade from treatment initiation could therefore contribute to longer-term disease control in a subset of patients, providing a plausible biological explanation for the later convergence of PFS curves.

Subgroup analyses according to baseline central nervous system involvement and EGFR mutation subtype did not show statistically significant differences in PFS between regimens. However, descriptive analyses based on PFS-RMST suggested that the absolute gain with Osi+CT compared with Ami+Laz was numerically greater in selected subgroups, including patients with baseline CNS metastases (approximately 1.3 months) and those harboring L858R mutations (approximately 2.1 months), compared with patients without CNS involvement or with exon 19 deletion. Although exploratory and not supported by formal statistical testing, these findings suggest that the magnitude of disease control over time with Osi+CT relative to Ami+Laz may vary across clinical and biological subgroups.

No differences in OS were observed between regimens at the current follow-up, likely influenced by post-progression therapies and subsequent treatment strategies. This underscores the need for more mature survival data and real-world evidence to better define the long-term clinical impact of first-line treatment approaches in EGFR-mutated NSCLC.

Safety remains a key determinant of treatment selection and clinical implementation. Osi+CT was associated with a higher incidence of grade ≥3 chemotherapy-associated toxicities, primarily hematologic and gastrointestinal, which are well known, predictable, and generally manageable with established supportive care measures familiar to most oncologists. In contrast, Ami+Laz was associated with a higher incidence of severe dermatologic adverse events, infusion-related reactions, and MET inhibition–related toxicities, such as hypoalbuminemia, peripheral edema, and thromboembolic events. These adverse events may require specific expertise and proactive management, implying an initial learning curve that could represent an early barrier to implementation in routine clinical practice.

Importantly, mitigation strategies have substantially improved the tolerability of Ami+Laz. The subcutaneous formulation of amivantamab markedly reduced infusion-related reactions compared with intravenous administration in the PALOMA-3 study (approximately 13% vs 66%) (25), while a dermatologic prophylaxis protocol—including oral doxycycline or minocycline, 1% clindamycin lotion for the scalp, 4% chlorhexidine for hand and foot hygiene, and a non-comedogenic ceramide-based moisturizer for the face and body—significantly decreased the incidence of grade ≥2 skin toxicity in the COCOON study (approximately 45% vs 75%), with fewer dose modifications and treatment discontinuations (26). These developments suggest that the tolerability profile of Ami+Laz may evolve with increasing clinical experience and optimized supportive care.

Several potential sources of bias inherent to cross-trial comparisons should be considered. In particular, the FLAURA trial enrolled a lower proportion of patients with baseline CNS metastases (~21%) compared with the more recent FLAURA-2 and MARIPOSA trials (~41%). However, the consistency of survival outcomes observed in the shared osimertinib arms suggests that this imbalance was unlikely to substantially affect the overall comparability of the trial populations. Differences in disease assessment protocols—such as mandatory serial brain imaging in MARIPOSA versus investigator-assessed imaging in earlier trials—may influence the detection and timing of progression events. Furthermore, variations in enrolment periods, crossover policies, and subsequent treatment landscapes may influence OS estimates.

Although no significant heterogeneity was observed in the shared osimertinib control arms, these trial-level differences may still introduce residual confounding. Accordingly, the numerical trends observed—such as the early separation of PFS curves favoring Osi+CT—should be interpreted as exploratory and hypothesis-generating, rather than as definitive evidence of superiority or equivalence. These findings may help inform clinical decision-making in the absence of head-to-head trials but should be interpreted in light of the potential for cross-trial bias until direct comparative evidence becomes available.

In conclusion, within the constraints of an indirect comparison, Osi+CT and Ami+Laz show no statistically significant differences in PFS, with point estimates and PFS-RMST suggesting numerically greater early disease control with Osi+CT. Differences in temporal patterns of disease control, resistance biology, toxicity profiles, and subgroup-specific RMST signals should be regarded as hypothesis-generating. In clinical practice, optimal treatment selection is therefore likely to depend on a careful balance between efficacy, toxicity, feasibility of management, physician experience, and—importantly—patient preferences, particularly when long-term disease control must be weighed against differing toxicity profiles.

## Data Availability

The raw data supporting the conclusions of this article will be made available by the authors, without undue reservation.
