# Correction: Efficacy and safety of first-line osimertinib monotherapy versus osimertinib plus platinum-based chemotherapy or amivantamab plus lazertinib in metastatic EGFR-mutated NSCLC: an indirect comparison

**DOI:** 10.3389/fonc.2026.1883614

**Published:** 2026-06-29

**Authors:** Luna Del Bono, Andrea Ossato, Lorenzo Gasperoni, Stefano Vecchia, Luca Cancanelli, Vera Damuzzo, Stefania Gori, Roberto Tessari, Teodoro Sava, Alessandro Inno

**Affiliations:** 1 School of Specialization in Hospital Pharmacy, Department of Pharmacy, University of Pisa, Pisa, Italy; 2 Territorial Pharmaceutical Service, Azienda Unità Locale Socio-Sanitaria (ULSS) 8 Berica, Vicenza, Italy; 3 Italian Society of Clinical Pharmacy and Therapeutics, Turin, Italy; 4 Pharmaceutical Department, Unità Sanitaria Locale (USL) Toscana Centro, Prato, Italy; 5 Hospital Pharmacy Unit, Ospedale Guglielmo da Saliceto, Piacenza, Italy; 6 Hospital Pharmacy Department, Azienda Ulss 2 Marca Trevigiana, Treviso, Italy; 7 Medical Oncology, Istituto di Ricovero e Cura a Carattere Scientifico (IRCCS) Ospedale Sacro Cuore Don Calabria, Negrar di, Valpolicella, Italy; 8 Hospital Pharmacy, Istituto di Ricovero e Cura a Carattere Scientifico (IRCCS) Ospedale Sacro Cuore Don Calabria, Negrar di, Valpolicella, Italy

**Keywords:** amivantamab, EGFR-mutated NSCLC, first-line therapy, indirect treatment comparison, lazertinib, network meta-analysis, osimertinib, reconstructed individual patient data

**Figures 2** and **4** were in the wrong order. Specifically, **Figure 2** and **Figure 4** were inadvertently interchanged in the original manuscript. The order has now been corrected. The correct order of figures and their captions are reported below.

**Figure 2:**
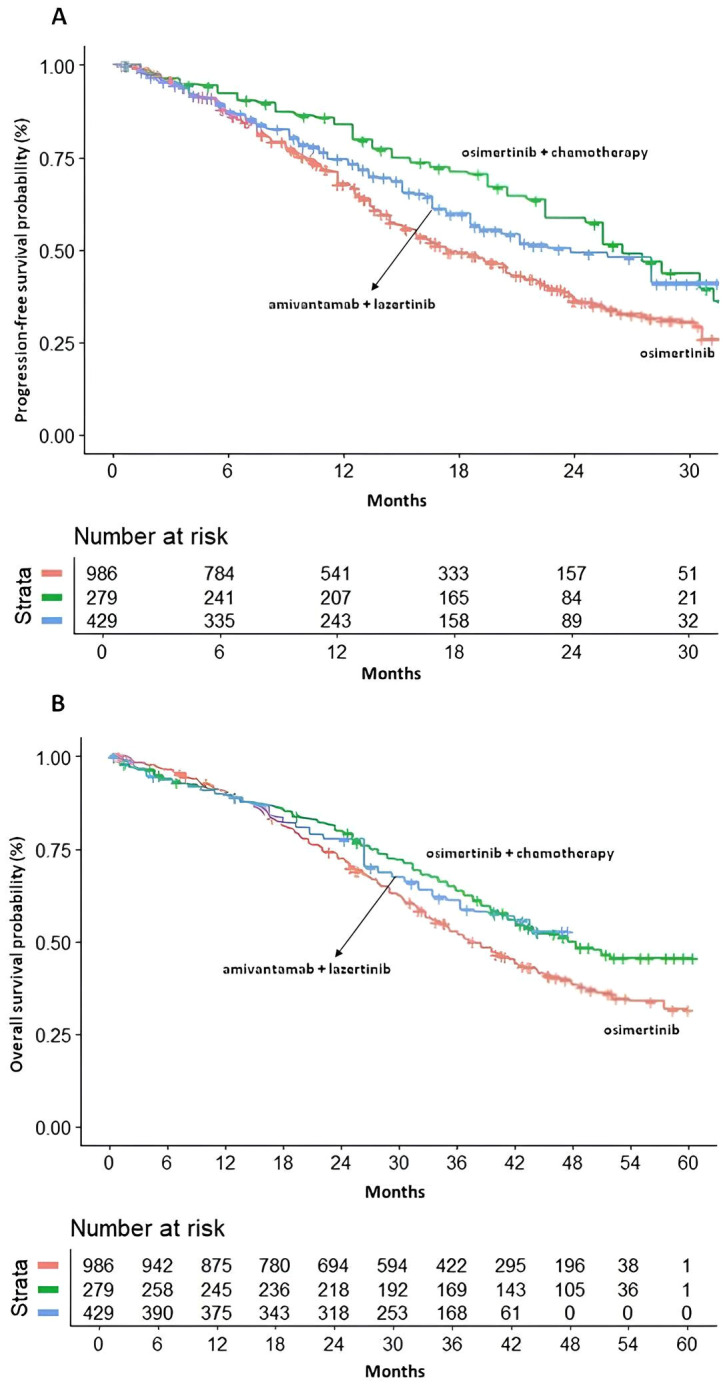
**(A)** PFS of first-line treatment options compared to osimertinib controls. After reconstruction of individual patient data from three trials, the following PFS KM curves were generated: Osi (n = 986; 3 cohorts (2, 12, 15); in red); Osi+CT (n = 279 from FLAURA 2 study (12); in green); and Ami+Laz (n = 429 from MARIPOSA study (15); in blue). **(B)** OS of first-line treatment options compared to osimertinib controls. After reconstruction of individual patient data from three trials, the following OS KM curves were generated: Osi (n= 986; 3 cohorts (3, 14, 16); in red); Osi+CT (n = 279 from FLAURA 2 study (14); in green); and Ami+Laz (n = 429 from MARIPOSA study (16); in blue). n, number of patients.

**Figure 4:**
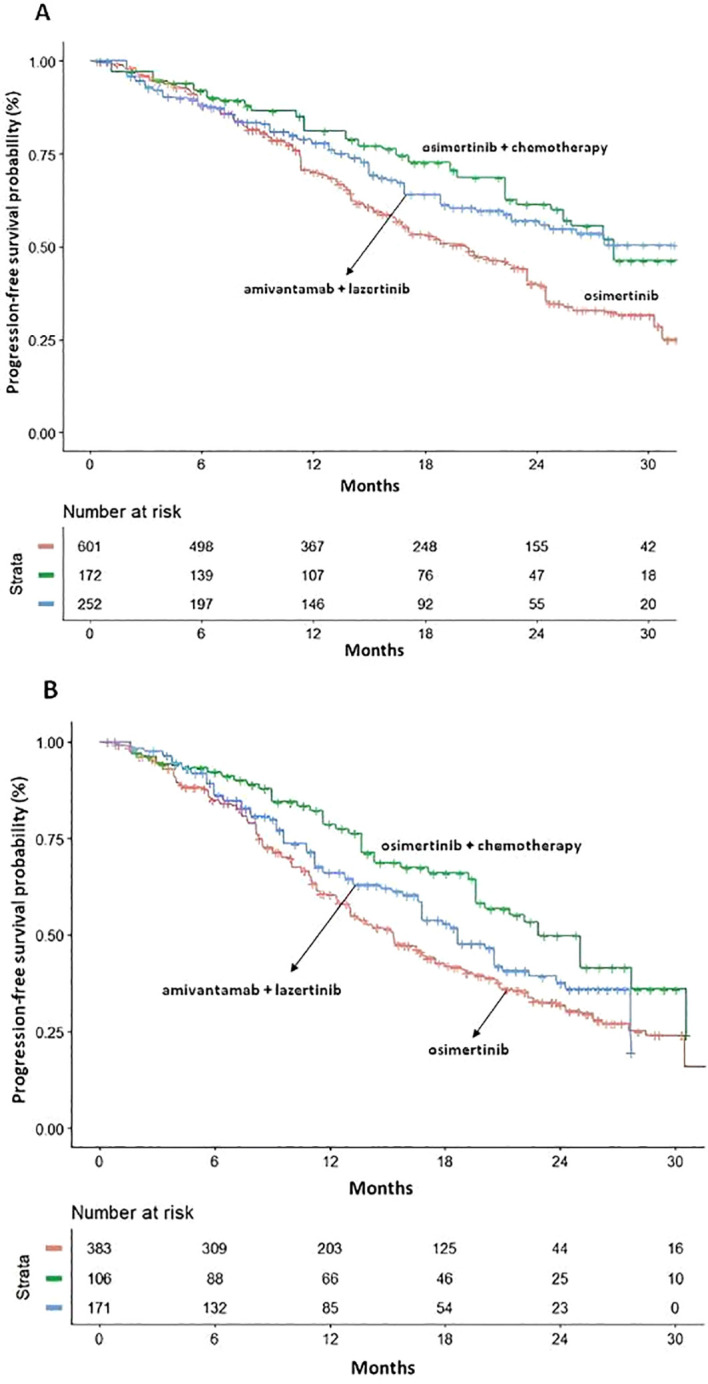
PFS of EGFRi in patients with Exon19 deletion **(A)** or with L858R mutation in exon 21 **(B)**. PFS KM curves are respectively reported: osimertinib (n=601 and n=383; 3 cohorts (2, 12, 15); in red); Osi+CT (n =172 and n=106 from FLAURA 2 study (12); in green); and Ami+Laz (n=252 and n=171 from MARIPOSA study (15); in blue). n, number of patients.

The original version of this article has been updated.

