# Supplementary material for: Efficacy and safety of first-line osimertinib monotherapy versus osimertinib plus platinum-based chemotherapy or amivantamab plus lazertinib in metastatic EGFR-mutated NSCLC: an indirect comparison
Source: Front Oncol. 2026 May 8;16:1802008. doi: 10.3389/fonc.2026.1802008 (PMC13193838; doi:10.3389/fonc.2026.1802008)
Supplement: Supplementary file 1 [file DataSheet1.docx]

Supplementary Material

# Supplementary Figures


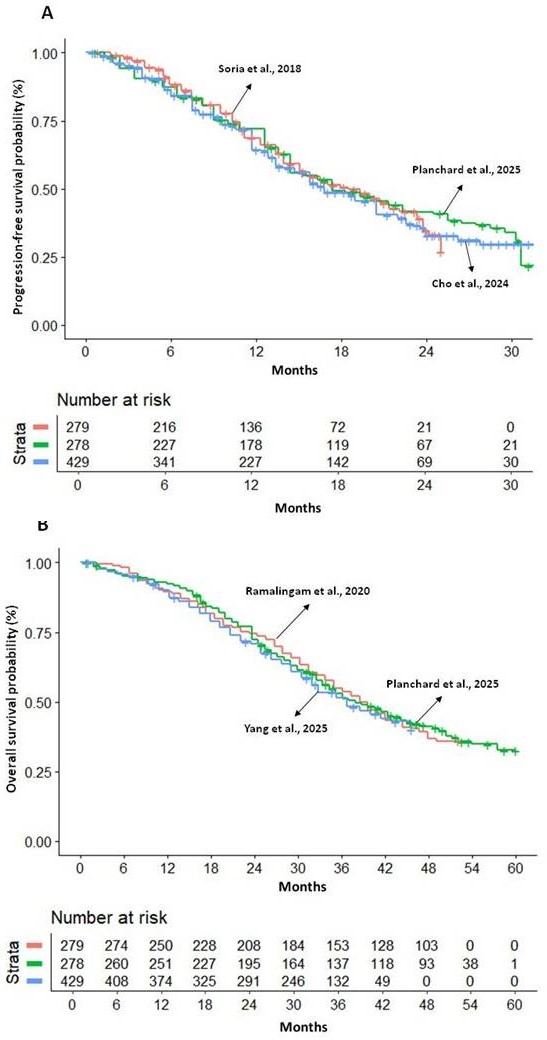


**Supplementary Figure 1.** Panel A: Kaplan–Meier curves of PFS generated after reconstructing patient-level data from the osimertinib-treated control arms of the included trials. FLAURA (n = 279; in red), FLAURA 2 (n = 278; in green), and MARIPOSA (n = 429; in light blue). Panel B: Kaplan–Meier curves of OS, generated after reconstructing patient-level data from the control arms of the included osimertinib-treated trials: FLAURA (n = 278; red) FLAURA 2 (n = 279; in green) and (n = 429; in light blue). Endpoint: progression-free survival (PFS), overall survival (OS) time in months. Abbreviations: n, number of patients.

*
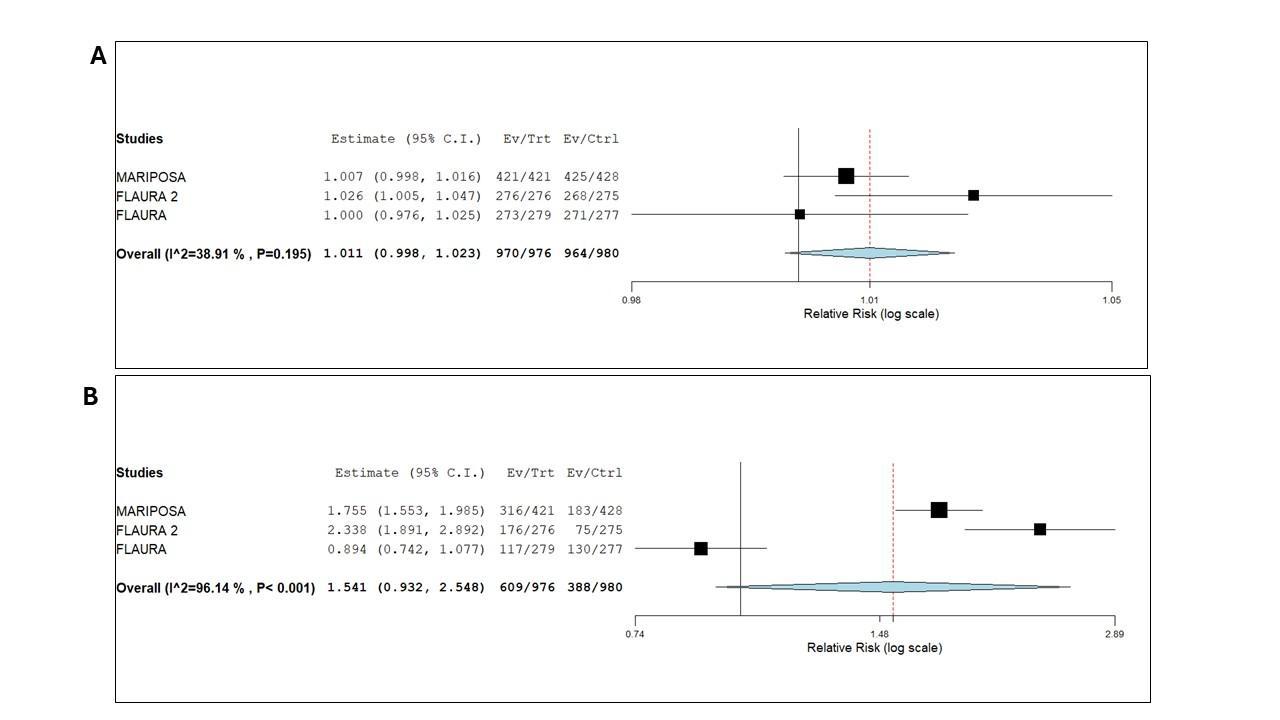
*

**Supplementary Figure 2.** Forest plot showing the risk ratio (RR) with 95% confidence interval (95%CI) for occurrence of any grade ADR (A) and grade ≥3 ADR (B) in patients treated with Ami+Laz (MARIPOSA Study), Osi+CT (FLAURA2 Study) and 1genEFR-TKI (FLAURA Study). Osi monotherapy serves as a reference treatment for comparison.
